# Unlocking the future of complex human diseases prediction: multi-omics risk score breakthrough

**DOI:** 10.3389/fbinf.2024.1510352

**Published:** 2024-12-16

**Authors:** Benson R. Kidenya, Gerald Mboowa

**Affiliations:** ^1^ Department of Biochemistry and Molecular Biology, Weill Bugando School of Medicine, Catholic University of Health and Allied Sciences, Mwanza, Tanzania; ^2^ Train-The-Trainers for Bioinformatics Group, Human Heredity and Health for Africa Bioinformatics Network (H3ABioNet), Cape Town, South Africa; ^3^ Department of Immunology and Molecular Biology, College of Health Sciences, School of Biomedical Sciences, Makerere University, Kampala, Uganda; ^4^ The African Center of Excellence in Bioinformatics and Data-Intensive Sciences, The Infectious Diseases Institute, College of Health Sciences, Makerere University, Kampala, Uganda; ^5^ Africa Centres for Disease Control and Prevention, African Union Commission, Addis Ababa, Ethiopia

**Keywords:** complex human disease prediction, multi-omics risk score, polygenic risk score (PRS), multi-omics, trait prediction

## Introduction

**FIGURE 1:**
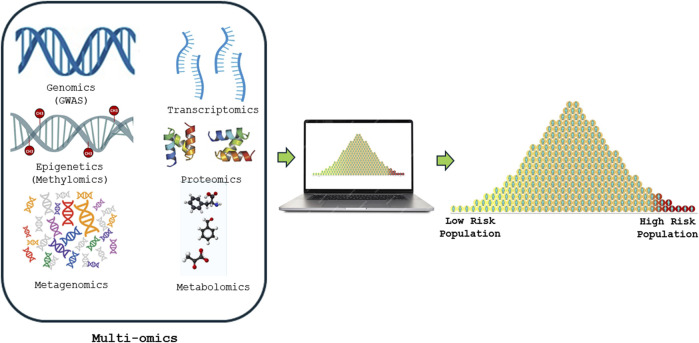
Shows how Multi-omics risk score is constructed from various high-throughput screening technologies such as genomics (GWAS), DNA methylomics, metagenomics, transcriptomics, proteomics and metabolomics to precisely predict and advancing the understanding of human diseases.

Precise prediction of the risk of acquiring complex human diseases using genomic data has gained a considerable traction among clinicians, medical geneticists and researchers, particularly in this era of next-generation sequencing. Multi-omics methods utilize various high-throughput screening technologies such as genomics (GWAS), DNA methylomics, metagenomics, transcriptomics, proteomics, metabolomics, and many others which play a crucial role in advancing the understanding of human diseases (Figure 1). These diverse multi-omics indicators create a comprehensive framework, yielding significant insights into future health status predictions. The polygenic risk scores (PRS) —a calculation of a person’s genetic predisposition to a trait or disease based on their genotype from pertinent genome-wide association study (GWAS) findings (Choi et al., 2020)— as well as methylation risk scores (MRS) —a linear combination of CpG (5′–C–phosphate–G–3′) methylation (covalent attachment of a methyl group onto the cytosine residue of DNA) states (Thompson et al., 2022)— have shown promise in predicting complex human diseases accurately (Liu et al., 2024). However, their translation into clinical care is yet to be realized. Several efforts have been made to improve their accuracy in predicting complex human diseases, such as increasing diversity in the genetic training databases, such as the *All of Us Research Program*, and including conventional risk factors in the PRS model (Liu et al., 2024). In the realm of predictive medicine, conventional risk factors span socio-demographic elements like age and sex, alongside anthropometric data such as body mass index (BMI) and crucial clinical measures, including blood pressure, lipid profiles, kidney and liver function tests, and other key biomarkers such as glycated hemoglobin (HbA1c). These conventional risk factors intertwine with lifestyle choices, behaviors, and environment.

The advent of GWAS, methylome-wide association studies (MWAS), and transcriptome-wide association studies (TWAS) have propelled genetic research forward by leaps and bounds, enabling the genotyping, methylation typing, and transcriptome analysis of millions of human samples. Through this vast endeavor, researchers have extracted genetic variants (Wu et al., 2021) and methylation patterns intricately linked to disease susceptibility across the human genome. The genetic variants and methylation patterns serve as the building blocks for constructing PRS (Choi et al., 2020) and MRS tailored to predict complex diseases in individuals based on their unique architecture. The efficacy and clinical potential of these tools shine brightly, offering invaluable insights into risk prediction for a plethora of common complex human diseases including cardiovascular diseases, cancers, diabetes mellitus, Alzheimer’s disease, and ankylosing spondylitis (Cappozzo et al., 2022). They represent transformative applications in the arsenal of personalized medicine, promising to revolutionize healthcare by unlocking more secrets hidden within our genomes.

The immense potential of genome-wide genotyping arrays lies in their ability to serve as a cost-effective approach capable of generating hundreds of PRSs. This groundbreaking technology is now undergoing rigorous evaluation in clinical studies across global healthcare systems. The allure of PRSs as predictive tools resonates profoundly, offering a glimpse into a future where personalized healthcare is not just a dream, but a tangible reality poised to transform medical practice. The full clinical potential of PRS and MRS remains largely untapped (Martin et al., 2019). This reality is especially pronounced in populations with high genetic diversity, diminished linkage blocks, and historical under-representation in genome databases, such as those of sub-Saharan African descent. The journey towards widespread clinical implementation of PRS is still in its infancy, with considerable challenges to overcome. Yet, with determination and concerted effort, bridging these gaps holds the key to unlocking the transformative power of PRS and MRS in diverse populations worldwide. Of note, there are concerted efforts such as *the All of Us Research Program* (Bick et al., 2024), Human Heredity and Health Africa (H3Africa), and others, to increase the representation of historically under-represented populations in the global genome databases to leverage this disparity. Ultimately the quantity and quality of data to compute PRS and MRS are escalating.

## Discussion

### The potential of multi-omics data in predicting precisely the human complex diseases

The advent of multi-omics technologies and accrued data thereof in recent era suggest the feasibility of measuring and combining various omics data and cellular factors. This enables the creation of multi-omics risk scores (MoRS) with enhanced predictability for complex diseases (Liu et al., 2024). PRS integrated with multi-omics data analyses, including metagenomics, epigenomics, and transcriptomics have revealed potential biomarkers and ultimately improved predictability for several prevalent age-related conditions like heart disease, diabetes, dementia, and various cancers (Liu et al., 2024).

The human gut microbiota, which refers to the collection of microorganisms residing in someone’s gastrointestinal tract, has been implicated in numerous common diseases (Chen et al., 2024; Huang et al., 2024). Specific microbial signatures in the gut have been linked to mortality and the development of diseases such as type 2 Diabetes (T2D), liver issues, and respiratory diseases among the general population (Liu et al., 2024). This suggests that the composition of the gut microbiome could potentially aid in predicting disease risk. It is worth noting that while GWAS has shed light on the genetic underpinnings of the gut microbiome, it is evident that the heritability of the gut microbiome is relatively low. Furthermore, similarities in the gut microbiome across generations are primarily associated with living in the same household rather than genetic factors. Recent studies have highlighted the association of various omics data such as gut metagenomics, DNA methylome data from epigenome-wide association studies and transcriptomics data with complex human diseases (Wu et al., 2021; Liu et al., 2024).

Recent research indicates that PRS models alone demonstrate superior predictive power compared to traditional risk factors (Liu et al., 2024). Furthermore, integrating MRS and transcriptomics data into the PRS showed a substantial improvement in prediction of prostate cancer (Wu et al., 2021). However, when conventional risk factors are incorporated into the PRS model, performance improves (Liu et al., 2024). Moreover, integrating both conventional risk factors and additional omics data such as from gut metagenomics into the PRS model significantly enhances its predictive performance for complex human diseases (Liu et al., 2024). Therefore, these studies demonstrated that the inclusion of other omics data from gut metagenomics, DNA methylomics and transcriptomics have shown a promise to improve the prediction of the incidence of age-related complex human diseases such as coronary artery disease, type 2 diabetes, Alzheimer’s disease, and prostate cancer (Wu et al., 2021; Liu et al., 2024).

Recent research suggests that studying blood DNA methylation at various CpG sites can serve as a valuable surrogate biomarker for exposure to risk factors, aiding in the prediction of complex human diseases such as cardiovascular diseases and in identifying high-risk populations (Cappozzo et al., 2022). Methylation risk scores (MRS) are typically constructed to model the relationship between methylation at CpG sites and specific traits or diseases through epigenome-wide association studies (EWAS). DNA methylation scores have been effectively utilized to assess an individual’s biological age (epigenetic clock) and have been strongly associated with several non-communicable diseases (NCDs) risk factors such as smoking, alcohol consumption, low physical activity, obesity, socioeconomic status, and occupational characteristics. This existing collinearity has made DNA methylation scores a powerful tool for predicting aging-related diseases as well as lifestyle-related diseases such as cancer and cardiovascular diseases (Cappozzo et al., 2022).

These epigenetic clocks demonstrate strong predictive capabilities for aging-related diseases and overall mortality. Research indicates that the risk of developing complex human diseases depends on the interaction between host genetic factors, environmental influences, and human behaviors or lifestyles. Incorporating conventional risk factors such as age, sex, smoking, and alcohol consumption into models accounts for human behavior (Levine et al., 2018). Studies, including the one conducted by Liu et al., have demonstrated that including these conventional risk factors improves the predictive ability for complex human diseases (Liu et al., 2024). Moreover, environmental factors, gene-environment interaction, and host lifestyle can be surrogated by epigenetic methylation analysis. Therefore, it is of the essence for the DNA methylomics data to be integrated into PRS models to enhance predictability for complex human diseases. There is a hypothesis suggesting that epigenomics data from DNA methylation might offer better predictive ability than many of the current PRS utilized today (Thompson et al., 2022). Consequently, integrating these multi-omics data into the PRS model could potentially yield the most effective predictive model for complex human diseases. To the best of our knowledge, there are very limited studies reported to integrate the DNA methylomics data into the PRS. Therefore, further investigations are warranted to explore the impact of integrating DNA methylomics data into PRS models for enhancing and predicting the development of complex human diseases.

Epigenetic modifications are widely recognized as influential factors in the biological pathways of both communicable diseases and non-communicable complex human diseases like hypertension and cancer with DNA methylation being the most extensively studied. Epigenetics involves the alteration of gene expression without changing the genetic code through processes such as DNA methylation and histone modification. This procedure entails attaching covalently a methyl group to the cytosine base found within sections containing repeated cytosine-guanine bonds, also referred to as CpG islands. When a gene undergoes heavy methylation, it typically remains transcriptionally silent (Irizarry et al., 2009). Environmental factors can trigger significant changes in methylation levels. The methylation patterns found in promoter CpG islands, which are clusters of CpG sites located in gene promoters, hold significant promise as potential biomarkers. They could play crucial roles in disease detection, disease classification, prognosis, and forecasting treatment responses (Ehrlich, 2019).

Recent research has revealed compelling links between DNA methylation and fluctuations in blood pressure, cardiovascular ailments, and various other non-communicable diseases. Han et al highlighted the pivotal role of gene-specific DNA methylation in elevating blood pressure, notably concerning factors like angiotensin-converting enzyme, lipid and amino acid metabolism, and impaired glucose metabolism (Han et al., 2016). Richard et al. pinpointed 13 replicated CpG sites, explaining 1.4% and 2.0% of individual differences in systolic and diastolic blood pressure, respectively (Richard et al., 2017). Intriguingly, new findings propose a strong link between DNA methylation and lifestyle choices (like smoking, alcohol intake, and diet), aging, obesity, and gender—all vital risk factors for hypertension. Kim et al. identified an association between DNA methylation in peripheral blood leukocytes and hypertension prevalence hints at the potential of DNA methylation as a high blood pressure biomarker (Kim et al., 2010). This highlights the promise of integrating DNA methylomics data into models to significantly enhance PRS performance.

### The most common bioinformatics and computational tools for multi-omics risk prediction of complex human diseased

Constructing multi-omics risk scores for complex human diseases typically involves integrating multiple layers of biological data (genomics, methylomics, metagenomics, transcriptomics, proteomics and metabolomics) into a single predictive score that quantifies disease risk. Bioinformatics and computational tools for construction multi-omics risk scores use various statistical, machine learning, and feature selection techniques to identify predictive markers across omics layers and combine them into a single aggregated risk score. Table 1 summarizes the most commonly used analytical tools for multi-omics data integration. By combining these bioinformatics and computational tools and frameworks, researchers can construct multi-omics risk scores that provide comprehensive, predictive insights into complex human disease susceptibility and progression. Ultimately enhancing our understanding of the molecular basis of these complex diseases by leveraging complementary information across multi-omics data.

**TABLE 1:** The most common bioinformatics and computational tools for multi-omics risk prediction of human complex diseased.

Category	Tool	Description [Rerefences]
Polygenic and Multi-Omics Risk Score Tools	PRSice	PRSice calculates PRS based on GWAS data. While primarily focused on genomics, PRSice can be used in combination with other omics tools to construct multi-omics risk scores by integrating genetic data with additional omics (Euesden et al., 2015)
	PRS-CS	PRS-CS is a Bayesian polygenic risk scoring tool that improves the accuracy of PRS by accounting for linkage disequilibrium via Bayesian regression and continuous shrinkage (CS) priors. This tool allows integrating genomics data with other omics layers, such as transcriptomics data, to build multi-omics risk scores (Ge et al., 2019)
Machine Learning and Deep Learning-Based Multi-Omics Tools	DeepOmics	A deep learning-based tool that integrates multi-omics data using neural networks to identify predictive biomarkers and generate risk scores. Its architecture can handle complex, non-linear relationships across omics layers, making it well-suited for multi-omics risk prediction (Kang et al., 2022)
	DeepOmics and MiBiOmics	These machine learning tools use various algorithms to integrate omics data and predict disease risk scores. They support feature selection, classification, and regression to create multi-omics risk prediction models (Zoppi et al., 2021; Ballard et al., 2024)
	MOFA+ (Multi-Omics Factor Analysis+)	It performs factor analysis to reduce dimensionality across omics datasets, identifying factors that contribute to disease risk. These factors can then be combined to build risk scores for predicting disease phenotypes and clinical outcomes (Argelaguet et al., 2020)
	CustOmics	CustOmics is a versatile deep-learning-based framework for multi-omics integration, designed for survival and classification tasks. It leverages customizable architectures to integrate data across omics types, particularly in cancer research (Benkirane et al., 2023)
Statistical and Probabilistic Tools for Multi-Omics Integration	PLIER (Pathway-Level Information ExtractoR)	PLIER is a tool for dimensionality reduction and feature selection that leverages known pathways to combine multiple omics layers. It can generate interpretable factors used in risk score modeling, making it ideal for multi-omics risk prediction (Mao et al., 2019)
	CIMLR (Consensus Independent Multilayer Learning)	CIMLR integrates multi-omics data by learning a consensus clustering across omics layers, useful for stratifying patients and creating disease risk scores. This method is effective in scenarios where disease subtypes must be identified in multi-omics datasets (Ramazzotti et al., 2018)
	Bayesian Omic Integrator (BOI)	BOI is a Bayesian framework that uses priors based on biological knowledge (e.g., pathway information) to integrate data across omics layers and predict risk. It is particularly effective in combining genomics and epigenomics data to improve disease risk prediction (Fang et al., 2018; Almutiri et al., 2024; Novoloaca et al., 2024)
Network-Based Tools for Multi-Omics Risk Scoring	NetDx	NetDx combines multi-omics data to build predictive patient similarity networks, which can be used to classify patient risk. NetDx allows for the creation of risk scores based on network-derived patient profiles and has been applied in cancer and psychiatric disease risk prediction (Pai et al., 2019)
	Mergeomics	Mergeomics uses network-based integration to link multi-omics markers with pathways and disease-related gene networks. By identifying critical network modules associated with disease risk, Mergeomics aids in building risk scores that combine multi-omics biomarkers (Shu et al., 2016)
Multi-Omics Risk Prediction Pipelines and Platforms	OmicsPipe	This end-to-end analysis pipeline provides workflows for analyzing multi-omics data, including RNA-seq, DNA-seq, and epigenomics data. OmicsPipe integrates these layers to build disease prediction models and can be adapted to produce multi-omics risk scores (Fisch et al., 2015)
	TranSMART	A translational research platform that integrates and analyzes multi-omics data, clinical data, and biomarker information. TranSMART includes tools for multi-omics data integration, risk score modeling, and data visualization. While tranSMART itself does not directly compute risk scores, it can help identify biomarker candidates and generate hypotheses about disease risk factors by linking omics data to clinical outcomes (Athey et al., 2013; tranSMART-Foundation/transmart, 2023)
Pathway and Functional Annotation Tools	MetaboAnalyst	Although primarily focused on metabolomics, MetaboAnalyst has multi-omics capabilities, including pathway analysis and functional annotation. It can identify metabolomics and gene expression biomarkers linked to disease risk. Researchers typically use MetaboAnalyst’s results in conjunction with statistical or machine learning models to develop personalized risk scores based on identified pathways and biomarkers (Pang et al., 2024)
	GeneMANIA	A network and pathway analysis tool that integrates multi-omics data, including genomics, transcriptomics, and proteomics information, to predict disease-related genes. GeneMANIA’s network-based approach can support risk score creation based on pathway associations (Mostafavi et al., 2008)
Integration and Visualization Tools	MixOmics	An R package that provides multi-omics integration and visualization methods, including PLS-DA, DIABLO, and multivariate factor analysis. It supports building predictive models and risk scores by selecting key features from multiple omics layers (Rohart et al., 2017)
	ComplexHeatmap	A visualization package in R that supports hierarchical clustering and multi-layered heatmaps for multi-omics data. It is commonly used to visualize relationships across omics layers, aiding in feature selection for risk scoring (Gu et al., 2016; Gu, 2022)

### Challenges, limitations and future research direction

The main challenges and limitations for multi-omics utility for improving prediction of complex human diseases are underrepresentation of diverse population in the genome databases, due to the fact that collecting multi-omics data is challenging due to the time consuming and costly process, particularly from large scale human genetic studies. As a result, multi-omics data may only be available for a subset of participants in a study limiting its statistical power, generalizability and hence transferability (Hasin et al., 2017; Chen and Han, 2023). Furthermore, lack of expertise among clinicians and biological scientists particularly from low income countries slows down the process to realize its clinical utility of multi-omics for all. Therefore, the concerted efforts are needed for establishment of multi-omics databases, as well as fostering training among biological scientists in health and life sciences in underserved areas.

Furthermore, several key limitations affect the reliability and effectiveness of multi-omics technologies. Batch effects are a significant challenge in omics data analysis, particularly in large-scale studies where samples are processed in batches or over extended periods. These technical variations between experimental runs can greatly impact data quality. Batch effects in omics studies refer to systematic differences in data caused by variations in experimental conditions across different batches of samples. These differences are unrelated to the biological variables or phenomena being studied and, if not properly addressed, can lead to misleading conclusions. Batch effects can arise from issues related to study design, such as flawed or confounded experimental setups or treatment effects. Variations in sample preparation and storage, including differences in protocols, reagents, equipment, storage conditions, operators, or laboratories, can also contribute to batch effects. Technical variations, such as inconsistencies in laboratory equipment, reagents, operators, or protocols across batches, further exacerbate the problem. Temporal variations, which occur when samples are processed at different times, and environmental factors, such as changes in temperature, humidity, or other conditions during sample preparation or analysis, are additional sources of variability (Yu et al., 2024).

High-throughput experiments are particularly prone to batch effects due to variability in data generated by different sequencing machines or mass spectrometry instruments. For example, DNA sequencing platforms may differ in kits, sequencing depth, quality, or laboratory practices. Bulk and single-cell RNA sequencing protocols may vary in terms of laboratory methods, RNA quality, or library size. Similarly, LC-MS-based proteomics and metabolomics can be influenced by differences in instruments, processing order, or laboratory-specific procedures (Yu et al., 2024). Finally, data analysis introduces its own challenges, with variability arising from differences in analysis platforms or pipelines, software tools, reference databases, and the treatment of low-detected or missing values. Addressing these sources of batch effects is critical to ensuring the reliability and reproducibility of multi-omics studies (Yu et al., 2024). Therefore, addressing batch effects is crucial to ensuring the reliability of results in multi-omics studies and minimizing the risk of drawing inaccurate conclusions.

Batch effects are notoriously common technical variations in multi-omics data and can lead to misleading outcomes if not properly addressed or if over-corrected. These effects can obscure true biological signals, create spurious associations, reduce the reproducibility and reliability of studies, and compromise the accuracy of downstream analyses, such as clustering, classification, or biomarker discovery. To address this challenge, several batch-effect correction algorithms have been developed to facilitate multi-omics data integration. However, their respective advantages and limitations must be thoroughly evaluated based on the type of omics data, performance metrics, and specific application scenarios before selecting an appropriate method for use (Ugidos et al., 2022; Yu et al., 2023; 2024). Assessing and mitigating batch effects is crucial to ensuring the reliability and reproducibility of omics data while minimizing the impact of technical variation on biological interpretation. As multi-omics data continue to expand, the importance of robust experimental design, optimized pipelines, and effective batch-effect correction algorithms is expected to grow, becoming central to large-scale research and clinical applications. The review by Yu et al. provides detailed insights into the sources, diagnostics, visualization techniques, and potential solutions for addressing batch effects in large-scale omics studies, including an overview of the currently available batch-effect correction algorithms (Yu et al., 2024). Reproducibility between individual studies is another significant concern, as experimental conditions can vary across studies, potentially leading to inconsistent results (Yu et al., 2023; Yu et al., 2024).

Additionally, certain omics types, such as transcriptomics, are particularly sensitive to sample extraction and preservation methods, which can introduce variability. Moreover, differences in platform technologies for the same omics type can further contribute to variability. For instance, DNA methylation analysis can be performed using a variety of BeadChip microarrays for detecting human DNA methylation. These include the *HumanMethylation27 BeadChip* (27K, which covers approximately 27,000 CpGs), *the HumanMethylation450 BeadChip* (450K, measuring over 485,000 CpGs), the *HumanMethylationEPIC BeadChip* (EPICv1 or 850K, measuring over 850,000 CpGs), and the *HumanMethylationEPIC v2.0 BeadChip* (EPICv2 or 900K, measuring over 900,000 CpGs (Lussier et al., 2024) as well as the gold standard, whole-genome bisulfite sequencing (WGBS), each offering different levels of resolution and coverage (Graw et al., 2021). Similarly, metabolomics can vary significantly depending on whether it is targeted or untargeted, presenting challenges in comparability. Lastly, there is the issue of interpreting absolute versus relative measures of risk, which complicates the translation of findings into practical applications (Canzler et al., 2020).

Furthermore, additional challenges include data complexity as integrating diverse data types with distinct scales, noise and missing values is challenging and requires advanced statistical and computational techniques, sophisticated normalization and imputation methods. Computational requirements are also a challenge as multi-omics data integration is computationally intensive, requiring high performance computing resources and efficient algorithms. Lastly, interpretability due to the complexity of multi-omics models that makes them challenging to interpret, and limiting their utility in clinical settings. The opportunity and future research direction of multi-omics risk prediction hinge at the fact that integrating multi-omics data within a systems biology framework fosters researchers to model pathways and networks that drive diseases, leading to insight into causative mechanisms and potential therapeutic target. Multi-omics risk prediction has the potential to tailor interventions based on an individual’s molecular profile, personalized medicine, optimizing prevention strategies and improving outcomes. This can be used in population preventive screening to identify high-risk individual for early intervention, particularly for diseases which early detection is critical such as cancer, cardiovascular diseases, diabetes mellitus, etc. Furthermore, the multi-omics has the potential to be used in understanding disease mechanisms by identifying the key molecular drivers of disease, it can highlight new therapeutic targets and inform drug development. Multi-omics risk prediction holds the promise for enhancing precision in complex human diseases risk assessment, with the potential to move to healthcare towards more individualized, proactive care. As methodologies and computational tools continue to advance, multi-omics integration will likely play an increasingly pivotal role in predicting, preventing and managing complex human diseases.

## Conclusion

In conclusion, human complex diseases arise from a complex interplay between the host genetics, host behaviors or lifestyle, and environmental factors. Integrating multi-omics data—such as metagenomics, epigenomics, particularly DNA methylomics, and transcriptomics data—and conventional risk factors into risk score models holds the potential for achieving the highest predictive performance for age-related complex human diseases compared to models based solely on PRS. Further research investigating the integration of multi-omics data is warranted to enhance PRS performance in predicting complex human diseases.

## References

[B1] Almutiri T. M. Alomar K. H. Alganmi N. A. (2024). Integrating multi-omics using bayesian ridge regression with iterative similarity bagging. Appl. Sci. 14, 5660. 10.3390/app14135660

[B2] Argelaguet R. Arnol D. Bredikhin D. Deloro Y. Velten B. Marioni J. C. (2020). MOFA+: a statistical framework for comprehensive integration of multi-modal single-cell data. Genome Biol. 21, 111. 10.1186/s13059-020-02015-1 32393329 PMC7212577

[B3] Athey B. D. Braxenthaler M. Haas M. Guo Y. (2013). tranSMART: an open source and community-driven informatics and data sharing platform for clinical and translational research. AMIA Jt. Summits Transl. Sci. Proc. 2013, 6–8. 24303286 PMC3814495

[B4] Ballard J. L. Wang Z. Li W. Shen L. Long Q. (2024). Deep learning-based approaches for multi-omics data integration and analysis. BioData Min. 17, 38. 10.1186/s13040-024-00391-z 39358793 PMC11446004

[B5] Benkirane H. Pradat Y. Michiels S. Cournède P.-H. (2023). CustOmics: a versatile deep-learning based strategy for multi-omics integration. PLoS Comput. Biol. 19, e1010921. 10.1371/journal.pcbi.1010921 36877736 PMC10019780

[B6] Bick A. G. Metcalf G. A. Mayo K. R. Lichtenstein L. Rura S. Carroll R. J. (2024). Genomic data in the all of us research Program. Nature 627, 340–346. 10.1038/s41586-023-06957-x 38374255 PMC10937371

[B7] Cappozzo A. McCrory C. Robinson O. Freni Sterrantino A. Sacerdote C. Krogh V. (2022). A blood DNA methylation biomarker for predicting short-term risk of cardiovascular events. Clin. Epigenetics 14, 121. 10.1186/s13148-022-01341-4 36175966 PMC9521011

[B43] Canzler S. Schor J. Busch W. Schubert K. Rolle-Kampczyk U. E. Seitz H. (2020). Prospects and challenges of multi-omics data integration in toxicology. Arch. Toxicol. 94 (2), 371–388. 10.1007/s00204-020-02656-y 32034435

[B8] Chen C. Han L. (2023). Multi-omic genetic scores advance disease research. Trends Genet. 39, 600–601. 10.1016/j.tig.2023.05.002 37295977 PMC10526975

[B9] Chen H. Ma Y. Xu J. Wang W. Lu H. Quan C. (2024). Circulating microbiome DNA as biomarkers for early diagnosis and recurrence of lung cancer. Cell Rep. Med. 5, 101499. 10.1016/j.xcrm.2024.101499 38582085 PMC11031421

[B10] Choi S. W. Mak T. S.-H. O’Reilly P. F. (2020). Tutorial: a guide to performing polygenic risk score analyses. Nat. Protoc. 15, 2759–2772. 10.1038/s41596-020-0353-1 32709988 PMC7612115

[B11] Ehrlich M. (2019). DNA hypermethylation in disease: mechanisms and clinical relevance. Epigenetics 14, 1141–1163. 10.1080/15592294.2019.1638701 31284823 PMC6791695

[B12] Euesden J. Lewis C. M. O’Reilly P. F. (2015). PRSice: polygenic risk score software. Bioinformatics 31, 1466–1468. 10.1093/bioinformatics/btu848 25550326 PMC4410663

[B13] Fang Z. Ma T. Tang G. Zhu L. Yan Q. Wang T. (2018). Bayesian integrative model for multi-omics data with missingness. Bioinformatics 34, 3801–3808. 10.1093/bioinformatics/bty775 30184058 PMC6223369

[B14] Fisch K. M. Meißner T. Gioia L. Ducom J.-C. Carland T. M. Loguercio S. (2015). Omics Pipe: a community-based framework for reproducible multi-omics data analysis. Bioinformatics 31, 1724–1728. 10.1093/bioinformatics/btv061 25637560 PMC4443682

[B15] Ge T. Chen C.-Y. Ni Y. Feng Y.-C. A. Smoller J. W. (2019). Polygenic prediction via Bayesian regression and continuous shrinkage priors. Nat. Commun. 10, 1776. 10.1038/s41467-019-09718-5 30992449 PMC6467998

[B44] Graw S. Chappell K. Washam C. L. Gies A. Bird J. Robeson M. S. 2nd (2021). Multi-omics data integration considerations and study design for biological systems and disease. Mol. Omics 17 (2), 170–185. 10.1039/d0mo00041h 33347526 PMC8058243

[B16] Gu Z. (2022). Complex heatmap visualization. iMeta 1, e43. 10.1002/imt2.43 38868715 PMC10989952

[B17] Gu Z. Eils R. Schlesner M. (2016). Complex heatmaps reveal patterns and correlations in multidimensional genomic data. Bioinformatics 32, 2847–2849. 10.1093/bioinformatics/btw313 27207943

[B18] Han L. Liu Y. Duan S. Perry B. Li W. He Y. (2016). DNA methylation and hypertension: emerging evidence and challenges. Briefings Funct. Genomics 15, 460–469. 10.1093/bfgp/elw014 27142121

[B19] Hasin Y. Seldin M. Lusis A. (2017). Multi-omics approaches to disease. Genome Biol. 18, 83. 10.1186/s13059-017-1215-1 28476144 PMC5418815

[B20] Huang Z. Huang X. Huang Y. Liang K. Chen L. Zhong C. (2024). Identification of KRAS mutation-associated gut microbiota in colorectal cancer and construction of predictive machine learning model. Microbiol. Spectr. 12, e0272023. 10.1128/spectrum.02720-23 38572984 PMC11064510

[B21] Irizarry R. A. Ladd-Acosta C. Wen B. Wu Z. Montano C. Onyango P. (2009). The human colon cancer methylome shows similar hypo- and hypermethylation at conserved tissue-specific CpG island shores. Nat. Genet. 41, 178–186. 10.1038/ng.298 19151715 PMC2729128

[B22] Kang M. Ko E. Mersha T. B. (2022). A roadmap for multi-omics data integration using deep learning. Brief. Bioinform 23, bbab454. 10.1093/bib/bbab454 34791014 PMC8769688

[B23] Kim M. Long T. I. Arakawa K. Wang R. Yu M. C. Laird P. W. (2010). DNA methylation as a biomarker for cardiovascular disease risk. PLoS One 5, e9692. 10.1371/journal.pone.0009692 20300621 PMC2837739

[B24] Levine M. E. Lu A. T. Quach A. Chen B. H. Assimes T. L. Bandinelli S. (2018). An epigenetic biomarker of aging for lifespan and healthspan. Aging (Albany NY) 10, 573–591. 10.18632/aging.101414 29676998 PMC5940111

[B25] Liu Y. Ritchie S. C. Teo S. M. Ruuskanen M. O. Kambur O. Zhu Q. (2024). Integration of polygenic and gut metagenomic risk prediction for common diseases. Nat. Aging 4, 584–594. 10.1038/s43587-024-00590-7 38528230 PMC11031402

[B26] Lussier A. A. Schuurmans I. K. Großbach A. Maclsaac J. Dever K. Koen N. (2024). Technical variability across the 450K, EPICv1, and EPICv2 DNA methylation arrays: lessons learned for clinical and longitudinal studies. Clin. Epigenetics 16, 166. 10.1186/s13148-024-01761-4 39578866 PMC11583407

[B27] Mao W. Zaslavsky E. Hartmann B. M. Sealfon S. C. Chikina M. (2019). Pathway-level information extractor (PLIER) for gene expression data. Nat. Methods 16, 607–610. 10.1038/s41592-019-0456-1 31249421 PMC7262669

[B28] Martin A. R. Kanai M. Kamatani Y. Okada Y. Neale B. M. Daly M. J. (2019). Clinical use of current polygenic risk scores may exacerbate health disparities. Nat. Genet. 51, 584–591. 10.1038/s41588-019-0379-x 30926966 PMC6563838

[B29] Mostafavi S. Ray D. Warde-Farley D. Grouios C. Morris Q. (2008). GeneMANIA: a real-time multiple association network integration algorithm for predicting gene function. Genome Biol. 9, S4. 10.1186/gb-2008-9-s1-s4 PMC2447538 18613948

[B30] Novoloaca A. Broc C. Beloeil L. Yu W.-H. Becker J. (2024). Comparative analysis of integrative classification methods for multi-omics data. Briefings Bioinforma. 25, bbae331. 10.1093/bib/bbae331 PMC11234228 38985929

[B31] Pai S. Hui S. Isserlin R. Shah M. A. Kaka H. Bader G. D. (2019). netDx: interpretable patient classification using integrated patient similarity networks. Mol. Syst. Biol. 15, e8497. 10.15252/msb.20188497 30872331 PMC6423721

[B32] Pang Z. Lu Y. Zhou G. Hui F. Xu L. Viau C. (2024). MetaboAnalyst 6.0: towards a unified platform for metabolomics data processing, analysis and interpretation. Nucleic Acids Res. 52, W398–W406. 10.1093/nar/gkae253 38587201 PMC11223798

[B33] Ramazzotti D. Lal A. Wang B. Batzoglou S. Sidow A. (2018). Multi-omic tumor data reveal diversity of molecular mechanisms that correlate with survival. Nat. Commun. 9, 4453. 10.1038/s41467-018-06921-8 30367051 PMC6203719

[B34] Richard M. A. Huan T. Ligthart S. Gondalia R. Jhun M. A. Brody J. A. (2017). DNA methylation analysis identifies loci for blood pressure regulation. Am. J. Hum. Genet. 101, 888–902. 10.1016/j.ajhg.2017.09.028 29198723 PMC5812919

[B35] Rohart F. Gautier B. Singh A. Cao K.-A. L. (2017). mixOmics: an R package for ‘omics feature selection and multiple data integration. PLoS Comput. Biol. 13, e1005752. 10.1371/journal.pcbi.1005752 29099853 PMC5687754

[B36] Shu L. Zhao Y. Kurt Z. Byars S. G. Tukiainen T. Kettunen J. (2016). Mergeomics: multidimensional data integration to identify pathogenic perturbations to biological systems. BMC Genomics 17, 874. 10.1186/s12864-016-3198-9 27814671 PMC5097440

[B37] Thompson M. Hill B. L. Rakocz N. Chiang J. N. Geschwind D. Sankararaman S. (2022). Methylation risk scores are associated with a collection of phenotypes within electronic health record systems. npj Genom. Med. 7, 50–11. Available at: https://github.com/tranSMART-Foundation/transmart (Accessed November 17, 2024). 10.1038/s41525-022-00320-1 36008412 PMC9411568

[B45] tranSMART-Foundation/transmart (2023). Dataset. Available at: https://github.com/tranSMART-Foundation/transmart.

[B38] Ugidos M. Nueda M. J. Prats-Montalbán J. M. Ferrer A. Conesa A. Tarazona S. (2022). MultiBaC: an R package to remove batch effects in multi-omic experiments. Bioinformatics 38, 2657–2658. 10.1093/bioinformatics/btac132 35238331 PMC9048667

[B39] Wu C. Zhu J. King A. Tong X. Lu Q. Park J. Y. (2021). Novel strategy for disease risk prediction incorporating predicted gene expression and DNA methylation data: a multi-phased study of prostate cancer. Cancer Commun. 41, 1387–1397. 10.1002/cac2.12205 PMC8696216 34520132

[B40] Yu Y. Mai Y. Zheng Y. Shi L. (2024). Assessing and mitigating batch effects in large-scale omics studies. Genome Biol. 25, 254. 10.1186/s13059-024-03401-9 39363244 PMC11447944

[B41] Yu Y. Zhang N. Mai Y. Ren L. Chen Q. Cao Z. (2023). Correcting batch effects in large-scale multiomics studies using a reference-material-based ratio method. Genome Biol. 24, 201. 10.1186/s13059-023-03047-z 37674217 PMC10483871

[B42] Zoppi J. Guillaume J.-F. Neunlist M. Chaffron S. (2021). MiBiOmics: an interactive web application for multi-omics data exploration and integration. BMC Bioinforma. 22, 6. 10.1186/s12859-020-03921-8 PMC7789220 33407076

